# The clinical value of acupuncture for women with premature ovarian insufficiency: a systematic review and meta-analysis of randomized controlled trials

**DOI:** 10.3389/fendo.2024.1361573

**Published:** 2024-07-11

**Authors:** Hengjie Cao, Huize Li, Guangyao Lin, Xuanling Li, Shimin Liu, Peiqi Li, Chao Cong, Lianwei Xu

**Affiliations:** ^1^ Department of Gynecology, Longhua Hospital, Shanghai University of Traditional Chinese Medicine, Shanghai, China; ^2^ School of Acupuncture-Moxibustion and Tuina, Shanghai University of Traditional Chinese Medicine, Shanghai, China

**Keywords:** premature ovarian insufficiency, acupuncture, randomized controlled trials, meta-analysis, review

## Abstract

**Objective:**

The aim of this study was to evaluate the therapeutic implications of acupuncture on improving ovarian function in women diagnosed with premature ovarian insufficiency (POI) through the implementation of randomized clinical trials (RCTs).

**Methods:**

A comprehensive search of eight databases was conducted to identify RCTs up until 5 October 2023. The outcomes included the levels of sex hormones, antral follicle count (AFC), Kupperman score, and total effective rate. The risk of bias (RoB) tool was utilized to evaluate the quality of the included studies. In order to guarantee the robustness and reliability of the findings, subgroup and sensitivity analyses were performed to investigate potential sources of heterogeneity.

**Results:**

A total of 13 RCTs comprising 775 patients were included in the study. Acupuncture demonstrated significant efficacy in reducing follicle-stimulating hormone (FSH) [SMD = 0.83, 95% CI (0.27, 1.39), *I* ^2 ^= 92%, *p* = 0.004], enhancing estradiol levels (E_2_) [SMD = 0.50, 95% CI (0.07, 0.93), *p* = 0.02, *I* ^2^ = 87%], and increasing anti-Müllerian hormone (AMH) [SMD = 0.24, 95% CI (0.05, 0.44), *p* = 0.01, *I* ^2^ = 8%], as well as improving the overall effective rate [RR = 1.22, 95% CI (1.10, 1.35), *p* < 0.01, *I* ^2^ = 14%]. Subgroup analysis revealed that compared with non-acupuncture therapy, the acupuncture with Chinese herbal medicine (CHM) and hormone replacement therapy (HRT) group exhibited a substantial reduction in FSH levels [SMD = 1.02, 95% CI (0.52, 1.51), *I* ^2^ = 60%, *p* < 0.01]. Furthermore, the acupuncture with CHM group also exhibited a substantial reduction [SMD = 4.59, 95% CI (1.53, 7.65), *I* ^2^ = 98%, *p* < 0.01]. However, only the acupuncture with CHM and HRT group demonstrated a significant increase in E_2_ levels [SMD = 0.55, 95% CI (0.23, 0.87), *I* ^2^ = 12%, *p <* 0.01].

**Conclusion:**

Acupuncture has demonstrated superiority over non-acupuncture in diminishing serum FSH levels and increasing serum E_2_, AMH, and the overall efficacy rate in women diagnosed with POI. These research findings suggest the necessity for broader-scale research with meticulous designs to fully demonstrate the efficacy and safety of acupuncture in the treatment of women with POI.

**Systematic review registration:**

https://www.crd.york.ac.uk, identifier CRD42023467751.

## 1 Introduction

Premature ovarian insufficiency (POI) is a syndrome characterized by ovarian hypofunction occurring prior to the age of 40, with an approximate incidence of 1% (1). Various factors including genetic (2, 3), immunological, viral, iatrogenic (4, 5), and environmental factors are common contributors to POI, with over 50% of patients encountering an etiology that remains undetermined (6). Irregular menstruation is a common manifestation in POI patients, presenting as oligomenorrhea or amenorrhea persisting for ≥4 months. The condition is characterized by elevated levels of gonadotropins and reduced estradiol, ultimately leading to a decrease in reproductive capacity. Symptoms of POI encompass hot flashes, perspiration, reduced libido, bone rarefaction, metabolic disturbances, and other repercussions. It not only impacts fertility, mental health, and quality of life but also exerts influence on various systems, including skeletal, cardiovascular, urogenital, and nervous systems among others (6–8). The concept of POI was introduced in 2008 (9), but its diagnosis has always lacked a precise criterion. In 2016, the European Society for Human Reproduction and Embryology (ESHRE) lowered the cutoff point for follicle-stimulating hormone (FSH) in early-onset ovarian insufficiency to 25 IU/L. This adjustment has drawn attention to POI, differentiating it from premature ovarian failure (POF).

Common treatments for POI encompass hormone replacement therapy (HRT), selenium and vitamin E supplementation, exercise therapy, and more (7). HRT is sequential estrogen–progesterone therapy with progesterone supplementation for 10 to 14 days per month in addition to continuous estrogen use. HRT stands as the recommended standard protocol for individuals with POI to alleviate symptoms of low estrogen (10, 11). Additionally, HRT has the potential to prevent cardiovascular diseases and bone rarefaction. However, this therapy has limitations as it cannot enhance ovarian activity, and breast cancer is a contraindication (12). Oral hormone therapy may elevate the risk of hypertension in women (13). Furthermore, judicious assessment is imperative for the use of HRT in POI patients with conditions such as SLE, gallbladder disorders, epilepsy, or asthma (6, 14, 15). A novel therapy known as *in-vitro* activation of follicles has been introduced, with a limited number of clinical pregnancy reports; however, its efficiency falls below the optimal level (16–18). Advanced treatments, including immunotherapy, stem cells, and gene editing, are currently in the research stage (19, 20).

Acupuncture, regarded as a traditional Chinese non-pharmacological intervention, has demonstrated promising outcomes, a high degree of safety, and minimal adverse effects. It is widely utilized in the field of reproductive endocrinology (21, 22). Recently, there has been a surge in randomized controlled trials investigating the efficacy of acupuncture for POI. It is imperative to integrate and systematically evaluate these research findings. Given the revised diagnostic criteria for POI by ESHRE in 2016 and the limited attention to the impact of acupuncture on the subset of POI patients with FSH >25 IU/L, a meta-analysis and systematic evaluation of existing data were conducted to furnish a pertinent reference for clinical practice.

## 2 Materials and methods

The Preferred Reporting Items for Systematic Reviews and Meta-Analyses guidelines (23) were followed in the reporting of this systematic review and meta-analysis (PROSPERO registration No. CRD42023467751).

### 2.1 Search strategy and study selection

Eight databases were comprehensively searched, namely, the English-language databases Cochrane Library, Web of Science, EMBASE, and PubMed and the Chinese-language databases Wanfang, VIP Information, CBM, and China National Knowledge Infrastructure (CNKI) from inception up to October 2023. Our retrieval strategy comprised three main components: clinical conditions (including premature ovarian failure, primary ovarian failure, primary ovarian insufficiency, premature ovarian insufficiency, premature menopause, POI, POF), interventions (such as acupuncture, electroacupuncture, manual acupuncture, warming needle, acupuncture therapy, needling, needles, needle therapy), and study types (RCT). No retrieval filters or limits were applied. To identify redundant papers, researchers manually examined the reference summaries of the retrieved articles. The initial screening of articles, independently conducted by the first two authors (H.J.C. and H.Z.L.), involved a thorough review of titles, abstracts, or full text to substantiate the eligibility of the studies. Any uncertainties regarding inclusion were deliberated among the other authors (L.W.X and S.M.L).

### 2.2 Inclusion and exclusion criteria

Studies that met the following criteria were included: 1) subjects: the standard of diagnosis was based on the clinical recommendations for the treatment of POI patients presented by ESHRE in 2016: a) women under 40 years old with amenorrhea/oligomenorrhea or symptoms of estrogen deficiency, b) oligomenorrhea or amenorrhea persisting for ≥4 months, and c) FSH level >25 IU/L on two occasions with a gap of >4 weeks; 2) intervention: acupuncture (including manual acupuncture and electroacupuncture regardless of the level of needling techniques), as well as the singular or combined use of Chinese herbal medicine or (and) HRT. Studies would be included if acupuncture was regarded as an adjuvant therapy for POI, and there were similar concomitant treatments between the experimental group and the control group; 3) controlled method: HRT, Chinese herbal medicine, or a combination of HRT and Chinese herbal medicine; 4) outcome indicators with sufficient data: effective rate, FSH, LH, E_2_, AMH, etc. Blood tests were performed before and after treatment, respectively, on the second to fourth days of the menstrual cycle to evaluate the basic hormone levels during the cycle; 5) study type: RCT; and 6) availability of complete data in the literature and precise data in the experimental and control groups.

Studies that met the following criteria were excluded: 1) interventions without acupuncture treatment (e.g., massage, moxibustion, or electrostimulation without needle); 2) interventions of control groups receiving different acupuncture treatments (e.g., acupoint catgut embedding); 3) patients suffering from other endocrine diseases (e.g., polycystic ovary syndrome, thyroid dysfunction, and hyperprolactinemia); 4) lack of definite or self-made criteria for efficacy evaluation; 5) studies about animal experiments, commentaries, editorials, experience introductions, conference articles, reviews, graduation theses, and case reports; 6) duplicate publication; 7) literature with incomplete outcome index data or full texts that cannot be obtained; and 8) literature with incorrect data and unidentified authors.

### 2.3 Data extraction and quality evaluation

Two authors, H.J.C. and H.Z.L., independently extracted relevant data using a standardized form. Information regarding the characteristics of the study population (such as sample size, age, and disease duration), treatment specifics (including types of interventions, acupoints, and duration), and group-wise results was collected.

Meanwhile, the quality assessment of the included studies was carried out by two independent reviewers (H.J.C. and H.Z.L.) utilizing the Cochrane Collaboration’s Risk of Bias tool. Any discrepancies were resolved through discussion with L.W.X.

The STRICTA (Standards for Reporting Interventions in Controlled Trials of Acupuncture: the STRICTA recommendations) (24) standard was used to evaluate acupuncture intervention measures. A response is considered “positive” by the STRICTA standard if every item is fully recorded. There were three categories for the reporting rate (*N* = reported RCTs/13): high (*N* ≥ 80%), moderate (*N* = 50%–80%), and low (*N* < 50%).

### 2.4 Statistical analysis

The data management software RevMan 5.3 was employed for data organization. Each group’s mean and standard deviation of the pretreatment and posttreatment results was collected. Continuous data were measured using the mean difference (MD) or standardized mean difference (SMD) with 95% confidence intervals (CIs). The following formula was used to figure out the *D*-value of the statistical mean and standard deviation: S * S = S1 * S1 + S2 * S2 − 2 * *R* * S1 * S2; *D* = M1 − M2 (*D*-value, difference before and after treatment; S, standard deviation of *D*-value; S1 and S2, standard deviation before and after treatment, respectively; M1 and M2, mean value before and after treatment; *R* = 0.4) (25). Statistical significance was established at *p <*0.05 on both sides. Dichotomous variables, such as the total effective rate, were presented as the risk ratio (RR). Additionally, *I* ^2^ statistics were utilized to assess interstudy heterogeneity. In cases of non-significant heterogeneity, we adopted the fixed-effects model; otherwise, the random-effects model was employed. A subgroup analysis based on the type of intervention was conducted to explore potential sources of heterogeneity. If a meta-analysis was considered inappropriate, we would offer a qualitative description of the results. To ensure result stability, a sensitivity analysis was performed by excluding specific studies. Moreover, if at least 10 studies were included, Begg’s tests and funnel plots were adopted by evaluating the *p*-value for publication bias.

## 3 Results

### 3.1 Included articles

**Figure 1:**
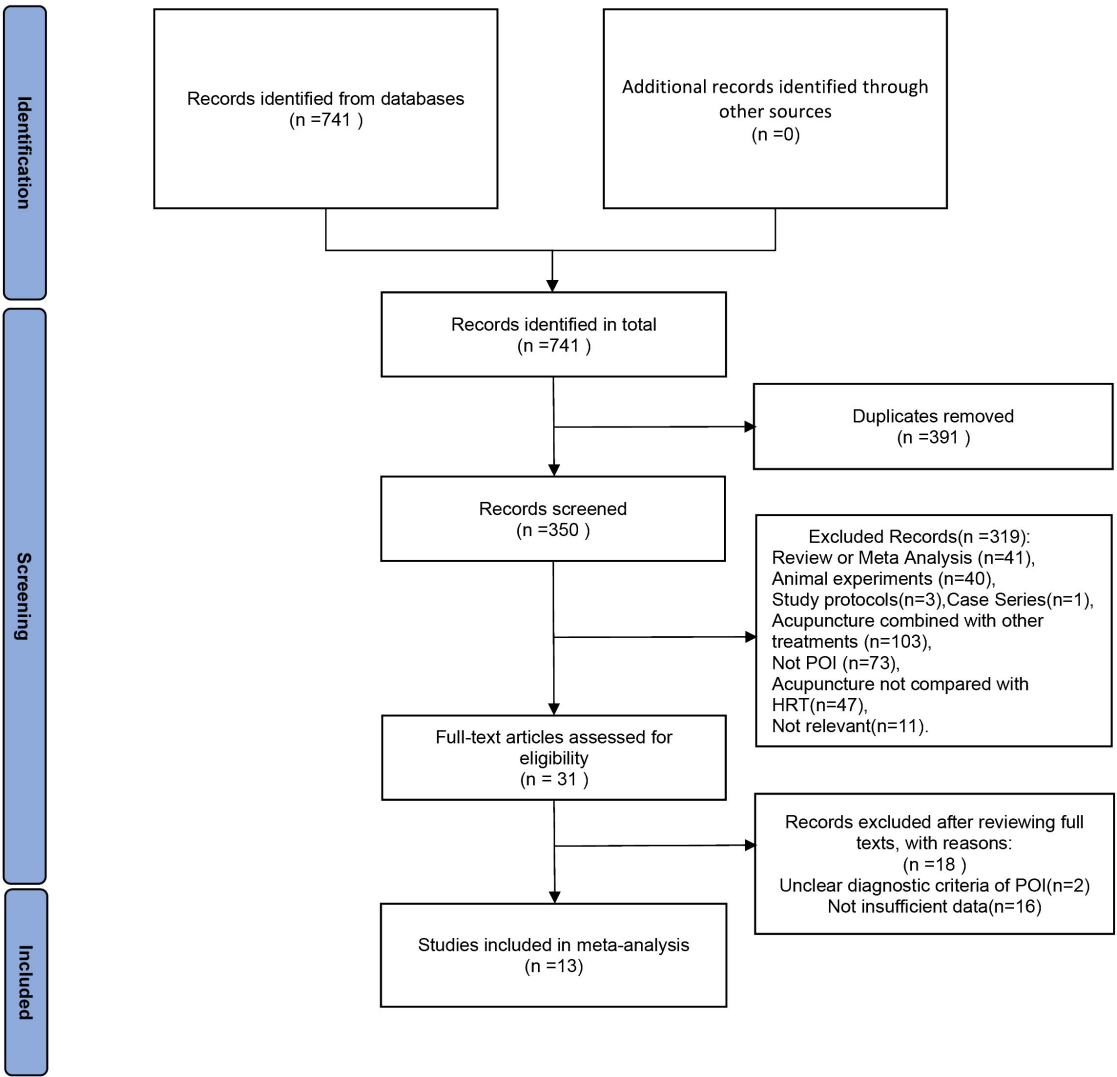
The PRISMA flowchart.

**Figure 1** illustrates the flowchart utilized for selecting publications. Initial database searches yielded 741 papers related to the therapeutic efficacy of acupuncture therapy in the treatment of premature ovarian insufficiency. After removing 391 duplicate publications, 350 pieces of literature remained. Upon reviewing the titles and abstracts among the remaining studies, 319 papers were excluded for failing to meet the inclusion criteria. Subsequently, 18 additional studies were excluded due to non-compliance with POI diagnostic criteria or insufficient data for evaluation. Finally, the meta-analysis included 13 RCTs published between 2019 and 2023.

### 3.2 Study characteristics

We incorporated 13 RCTs, all conducted in China and published in Chinese between 2019 and 2023. The studies encompassed 775 POI patients, divided into an experimental group (acupuncture group) and a control group, with 388 and 387 cases, respectively. In the studies, blood samples were collected on days 2–4 of the menstrual cycle to assess baseline sex hormone indices. Twelve of the studies collected blood samples before and after the last treatment, while the other study (26) collected blood samples before treatment and 3 months after the cessation of treatment, respectively. Among them, 13 reported FSH levels (26–38), 12 reported LH and E_2_ levels (26–33, 35–38), 8 reported AMH levels (26, 27, 31–33, 35–37), 5 trials reported the total effective rate (26, 30–32, 35), and 9 presented adverse events (26, 27, 29–33, 35, 38). Baseline homogeneity was observed across all RCTs. Comprehensive details on the characteristics of the study are shown in **Table 1**.

**Table 1:** Study characteristics.

Study	Year	Sample size (*n*)	Age (years)		Disease duration (months)		Treatment regimen		Treatment frequency	Treatment duration (months)	Outcomes
		T/C	T	C	T	C	T	C			
Bai, (38)	2023	30/30	32.6 ± 5.8	30.8 ± 5.5	17.8 ± 7.3	16.1 ± 6.2	Acu.	HRT	5 times a week	3	①②③④⑤⑥
Liang, (37)	2022	32/30	33.56 ± 4.73	33.50 ± 4.69	NA	NA	Acu.+CHM+HRT	CHM+HRT	3 times a week	3	①②③
Zhuo, (36)	2021	40/40	34.52 ± 3.12	35.78 ± 3.27	15.6 ± 2.9	16.2 ± 3.3	Acu.+HRT	HRT	3 times a week	3	①②③④
Xu Qing, (35)	2021	30/30	33.90 ± 4.57	32.87 ± 4.86	11.07 ± 6.53	13.17 ± 6.61	Acu.+CHM	CHM	2 times a week	3	①②③④⑦
Xu Chengchao, (34)	2021	30/30	31 ± 4	29 ± 5	14.5 ± 6.0	12.2 ± 4.4	Acu.	HRT	5 times a week	3	①⑤
Liu, (33)	2021	21/22	34.36 ± 5.18	34.44 ± 5.92	20.52 ± 14.07	18.18 ± 10.37	Acu.	HRT	3 times a week	3	①②③④⑤⑥
Hui, (26)	2021	30/30	34 ± 3	34 ± 4	14.30 ± 2.36	14.25 ± 2.32	Acu.+HRT	HRT	3 times a week	3	①②③④⑥⑦
Zhang, (32)	2020	30/30	35.16 ± 3.32	36.50 ± 3.22	12.80 ± 9.03	10.86 ± 9.52	Acu.+CHM+HRT	CHM+HRT	3 times a week	3	①②③④⑦
Song, (31)	2020	30/30	34.60 ± 3.78	35.33 ± 2.99	13.00 ± 10.99	12.10 ± 10.08	Acu.+CHM+HRT	CHM+HRT	3 times a week	3	①②③④⑦
Qiu, (30)	2020	29/29	31.90 ± 4.21	32.24 ± 4.56	11.25 ± 5.56	10.89 ± 4.07	Acu.+HRT	HRT	3 times a week	3	①②③⑤⑦
Zhang, (29)	2019	25/25	31 ± 4	33 ± 4	19.2 ± 10.8	19.2 ± 9.6	Acu.	HRT	3 times a week	3	①②③
Qi, (28)	2019	30/30	31.15 ± 4.84	29.15 ± 4.86	40.56 ± 13.8	42 ± 14.52	Acu.+CHM	CHM	3 times a week	3	①②③⑥
Miao, (27)	2019	31/31	34.58 ± 0.76	34.10 ± 0.83	23.06 ± 2.18	22.39 ± 2.17	Acu.+CHM	CHM	3 times a week	3	①②③④

T, trial group; C, control group; NA, not available; Acu., acupuncture; CHM, Chinese herbal medicine; HRT, hormone replacement therapy; ① follicle-stimulating hormone (FSH); ② luteinizing hormone (LH); ③ estradiol (E_2_); ④ anti-Müllerian hormone (AMH); ⑤ Kupperman score; ⑥ antral follicle count (AFC); ⑦ total effective rate.

### 3.3 Quality assessment

**Figure 2:**
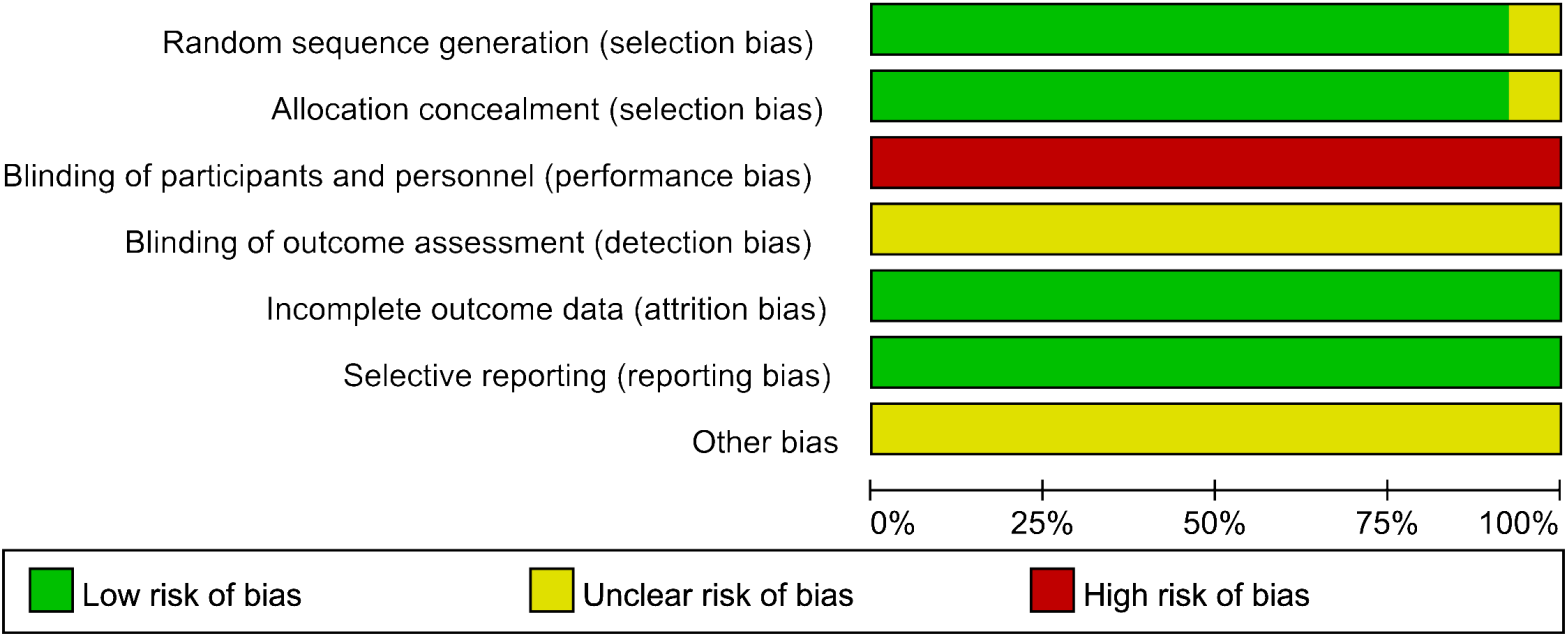
Risk of bias assessment.

Except for Song (31), the methodological quality of all studies regarding selection bias was assessed as low risk due to clear procedures and concealed allocation in the patient randomization process. Given the inherent limitation that acupuncture therapy cannot be blinded, all included RCTs were categorized as “high risk” for participant and staff blinding. Incomplete outcome data and selective reporting were considered to pose a low risk of bias across all studies. Among the 13 studies, no indication of potential bias was identified (**Figure 2**). The quality of interventions reported was evaluated against the STRICTA list, with a mean reporting rate of 71.04% for all entries (**Table 2**). In summary, the quality of the included studies was considered moderate.

**Table 2:** Quality evaluation based on the STRICTA list.

Items	Item details		Reported RCTs	%
1. Acupuncture rationale	1a	Style of acupuncture	13	100%
	1b	Reasoning for treatment provided	13	100%
	1c	Extent to which treatment was varied	0	0%
2. Details of needling	2a	Number of needle insertions per subject per session	13	100%
	2b	Names of points used	13	100%
	2c	Depth of insertion	11	85%
	2d	Response sought	8	62%
	2e	Needle stimulation	12	92%
	2f	Needle retention time	13	100%
	2g	Needle type	13	100%
3. Treatment regimen	3a	Number of treatment sessions	13	100%
	3b	Frequency and duration of treatment sessions	13	100%
4. Other components of treatment	4a	Details of other interventions administered to the acupuncture group	5	38%
	4b	Setting and context of treatment	3	23%
5. Practitioner background	5	Description of participating acupuncturists	0	0%
6. Control or comparator interventions	6a	Rationale for the control or comparator in the context of the research question, with sources that justify this choice	13	100%
	6b	Precise description of the control or comparator	13	100%

### 3.4 Outcome measurements

#### 3.4.1 FSH levels

**Figure 3:**
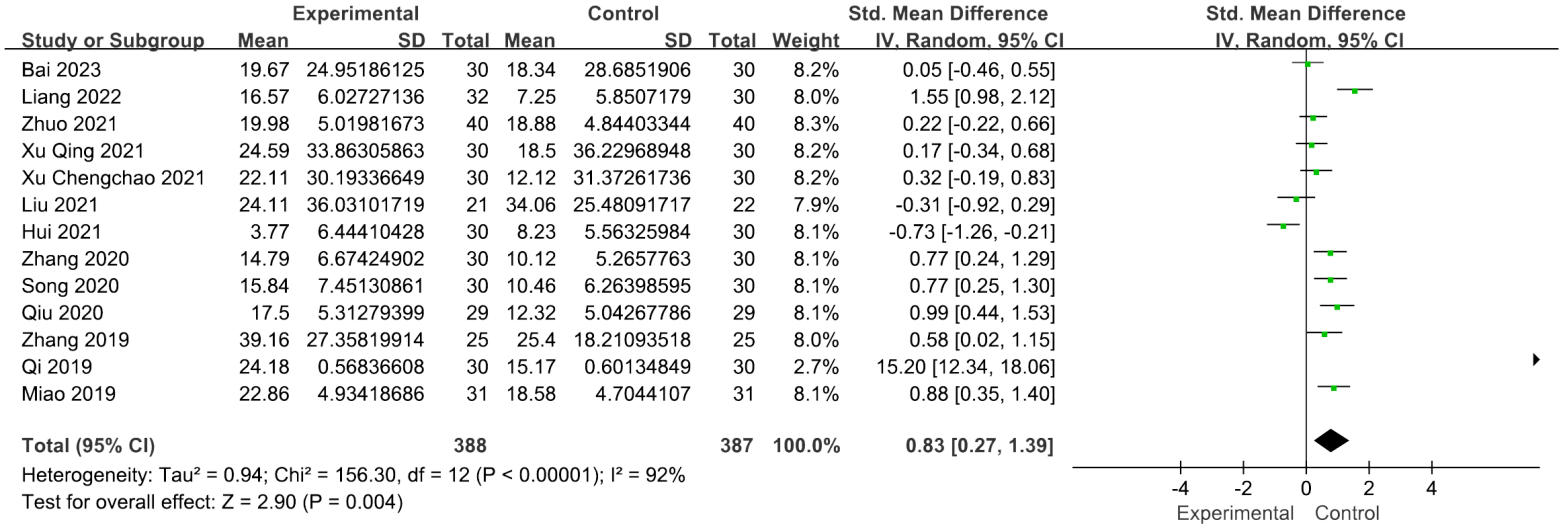
Forest plot illustrating the relationship between FSH levels and acupuncture therapy.

Thirteen studies reported FSH levels of 388 participants in the acupuncture group. Combining the results of these studies showed that acupuncture dramatically reduced the levels of FSH in women with POI [SMD = 0.83, 95% CI (0.27, 1.39), *I* ^2^ = 92%, *p* = 0.004] (**Figure 3**). Sensitivity analysis was employed to confirm the robustness of the aggregated outcomes.

#### 3.4.2 LH levels

**Figure 4:**
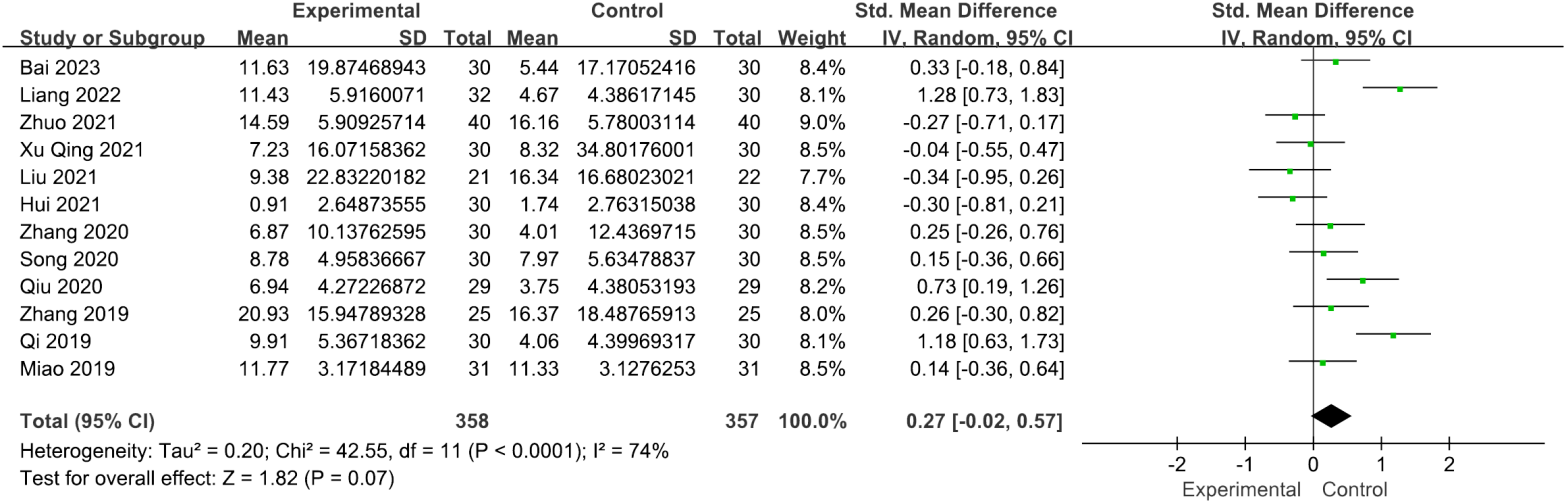
Forest plot illustrating the relationship between LH levels and acupuncture therapy.

Twelve studies involving 715 patients focused on the LH levels. Compared with the control groups, the acupuncture group had no advantage [SMD = 0.27, 95% CI (−0.02, 0.57), *I* ^2^ = 74%, *p* = 0.07] (**Figure 4**). The robustness of the combined findings was confirmed through sensitivity analysis.

#### 3.4.3 Estradiol levels

**Figure 5:**
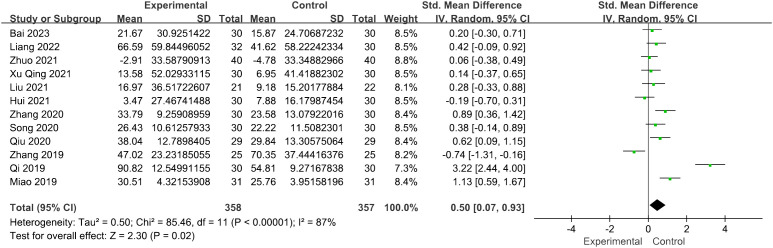
Forest plot illustrating the relationship between E_2_ levels and acupuncture therapy.

The evaluation of the impact of acupuncture on estradiol levels yielded 12 RCTs with a total of 715 individuals. It showed a statistically significant connection between the function of acupuncture and the improvement in estradiol levels [SMD = 0.50, 95% CI (0.07, 0.93), *I* ^2^ = 87%, *p* = 0.02] (**Figure 5**). The pooled estimates remained unaffected by any individual study, as confirmed through the sensitivity analysis.

#### 3.4.4 AMH levels

**Figure 6:**
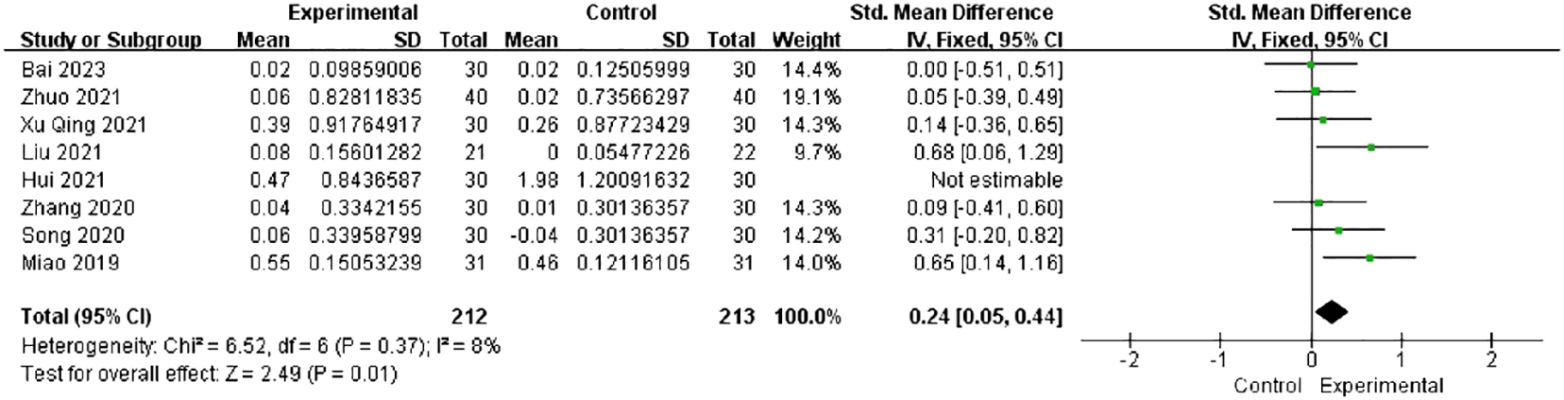
Forest plot illustrating the relationship between AMH levels and acupuncture therapy.

Among all studies, only eight studies reported the AMH levels. The heterogeneity dropped from 81% to 8% after the exclusion of one study (26) in the sensitivity analysis. The combined findings of seven trials with 212 individuals showed a significant increase in the AMH levels [SMD = 0.24, 95% CI (0.05, 0.44), *I* ^2^ = 8%, *p* = 0.01] (**Figure 6**).

#### 3.4.5 KI score

**Figure 7:**
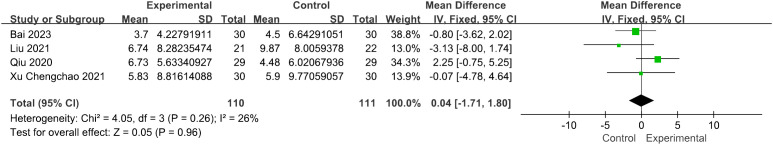
Forest plot illustrating the relationship between KI score and acupuncture therapy.

Four studies reported the modified KI score (39). The results of the fixed-effects model analysis indicated that there was no statistically significant difference between the control group and the acupuncture therapy group [MD = 0.04, 95% CI (−1.71, 1.80), *I* ^2^ = 26%, *p* = 0.96] (**Figure 7**).

#### 3.4.6 Antral follicle count

**Figure 8:**
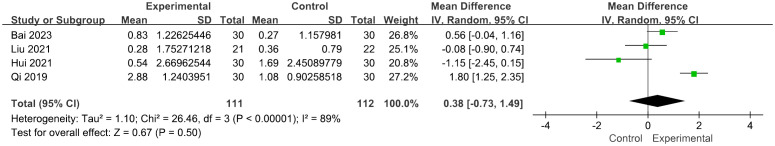
Forest plot illustrating the relationship between AFC and acupuncture therapy.

This meta-analysis of the antral follicle count (AFC) result contained 111 participants from a total of four studies. The pooled result revealed that the effects of acupuncture did not exhibit a significant difference from others [MD = 0.38, 95% CI (−0.73, 1.49), *I* ^2^ = 89, *p* = 0.50] (**Figure 8**). Furthermore, following the sensitivity analysis, the outcomes remained unchanged.

#### 3.4.7 Total effective rate

**Figure 9:**
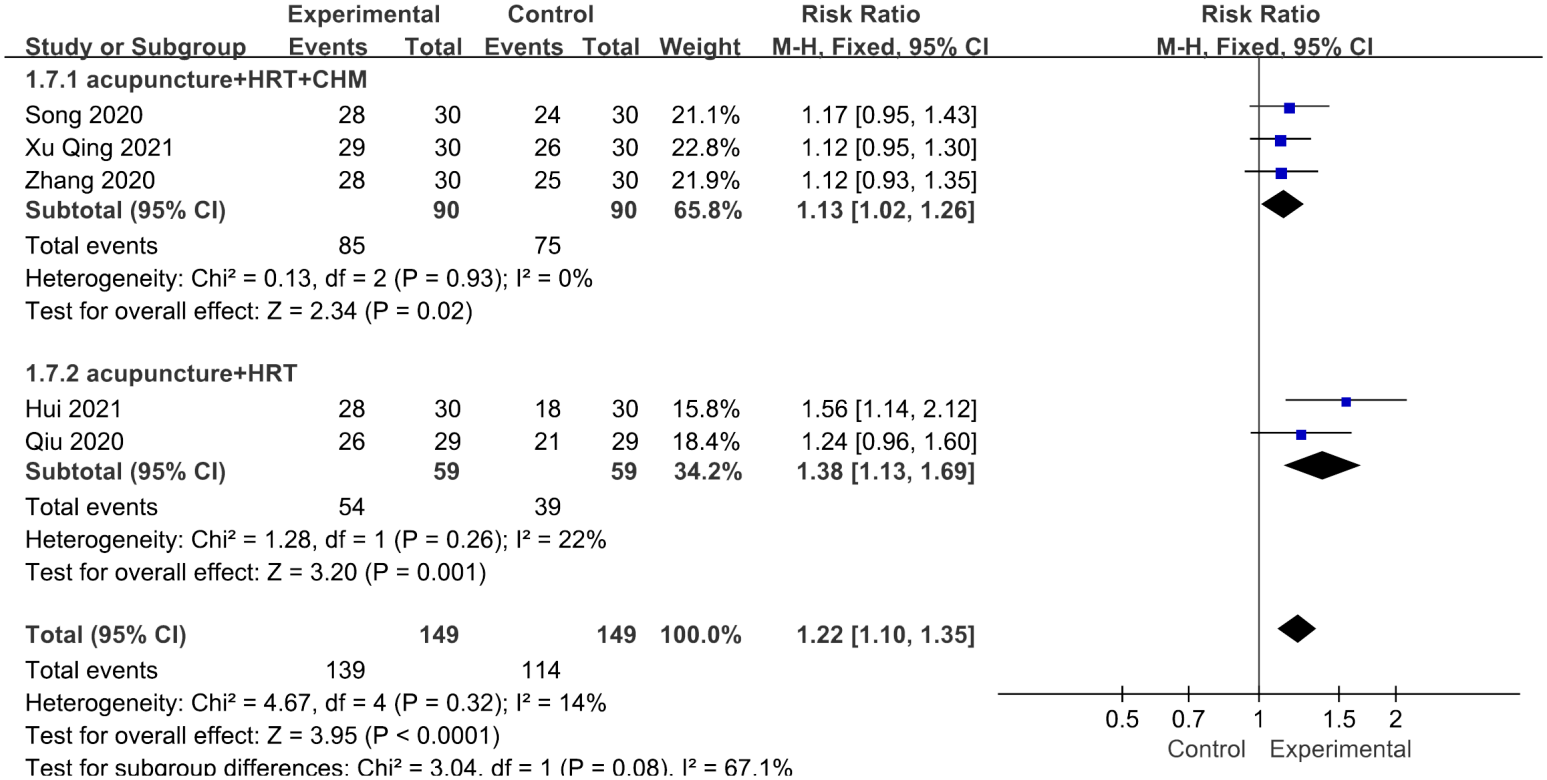
Forest plot illustrating the relationship between total effective rate and acupuncture therapy.

Five studies assessed the overall effective rate (26, 30–32, 35), sticking to the same score scale including the menstrual cycle, menstrual blood volume, and general symptoms, such as palpitation and sleeplessness. Pretreatment and posttreatment evaluations were conducted, revealing a notable increase of 30% or more in symptom amelioration, thereby indicating effectiveness. The study results indicated that patients who received acupuncture treatment had a higher overall effective rate compared with those who did not receive acupuncture treatment [RR = 1.22, 95% CI (1.10, 1.35), *I* ^2^ = 14%, *p* < 0.01] (**Figure 9**). The figure revealed low heterogeneity.

#### 3.4.8 Adverse effect

**Figure 10:**
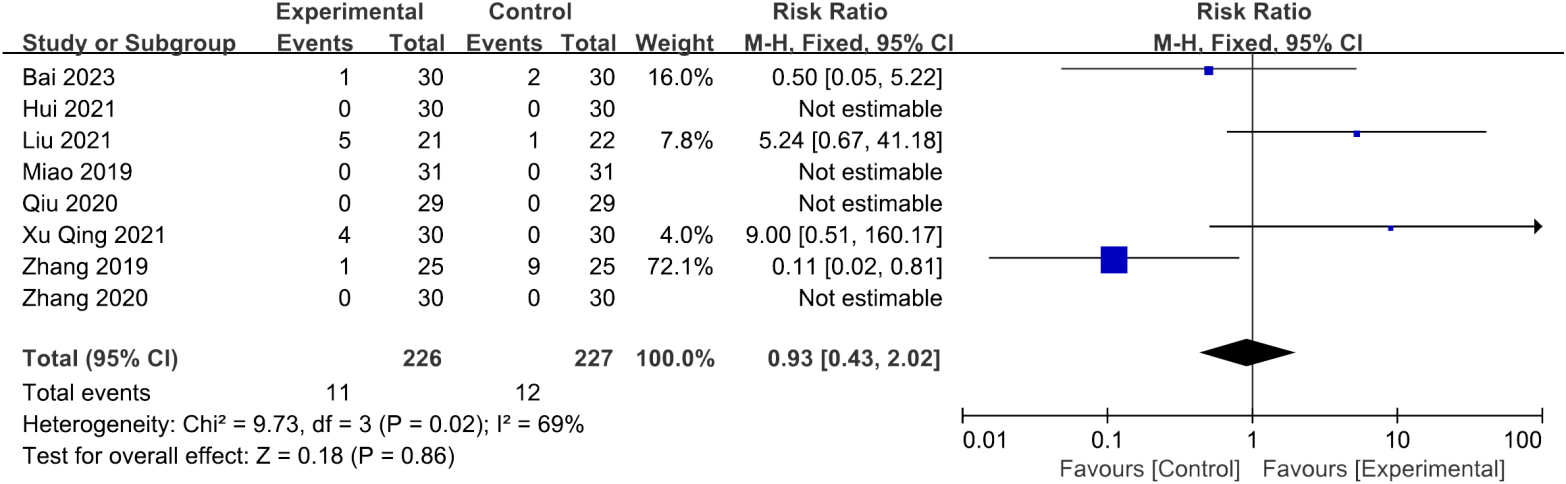
Forest plot illustrating the relationship between adverse effect and acupuncture therapy.

Nine of the thirteen studies reported the situation of adverse effects: five of them reported adverse effects (29, 31, 33, 35, 38), while the remaining four documented no adverse effects (26, 27, 30, 32). One study reporting abdominal distension was excluded due to insufficient detailed description (31), while the following statistics included eight studies (26, 27, 29, 30, 32, 33, 35, 38). Among 226 patients in the trial groups, 11 cases of adverse events were reported, consisting of nine cases of subcutaneous hemorrhage (33, 35, 38), one case of breast distending pain (29), and one case of needle sticking (35). In the control groups, 12 adverse events were reported in 227 patients, consisting of five cases of stomach discomfort (29, 33), five cases of breast distending pain (29), and two episodes of menostaxis (38). Meta-analysis showed that the difference between the two groups lacks statistical significance (*p* = 0.86 > 0.05) (**Figure 10**).

### 3.5 Subgroup analysis

Due to three pooled results indicating significant heterogeneity (*I* ^2^ > 60%) in the levels of FSH, LH, and estradiol, subgroup analysis was conducted based on various types of interventions.

#### 3.5.1 FSH and LH levels

**Figure 11:**
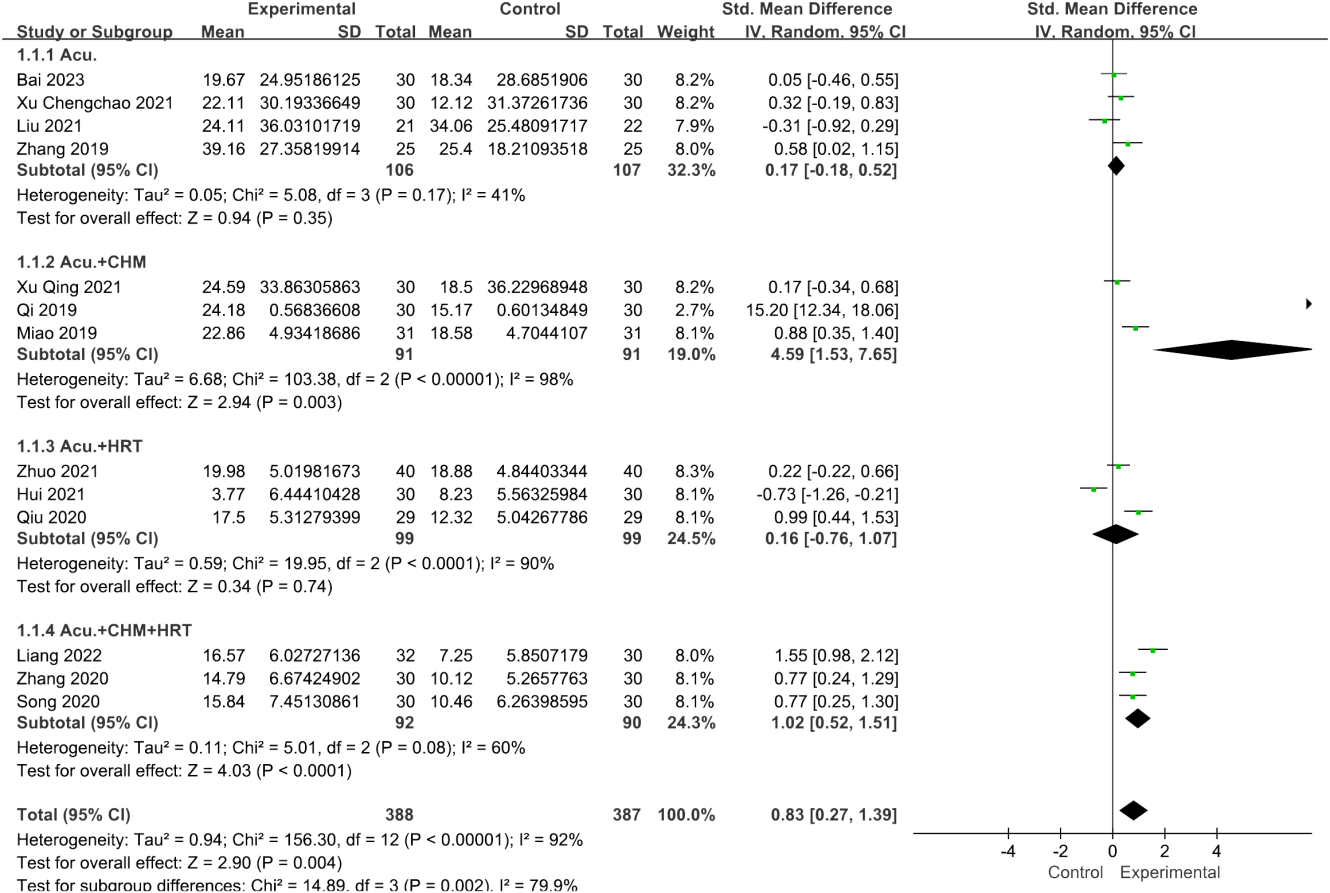
Forest plot illustrating the relationship between FSH levels and acupuncture therapy with subgroup analysis.

**Figure 12:**
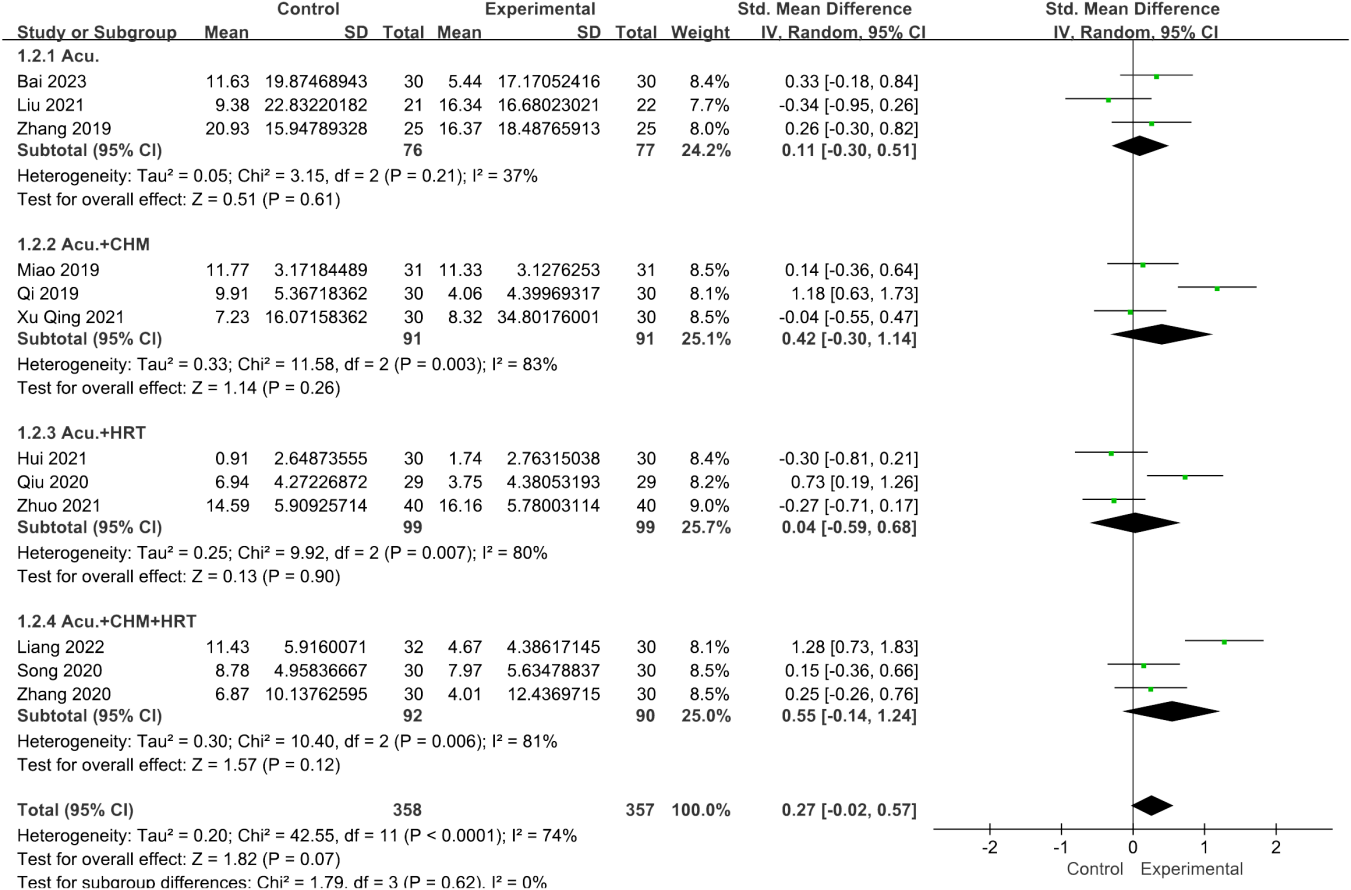
Forest plot illustrating the relationship between LH levels and acupuncture therapy with subgroup analysis.

In the subgroup analysis for FSH levels, the pooled result revealed that both the acupuncture combined with CHM and HRT group [SMD = 1.02, 95% CI (0.52, 1.51), *I* ^2^ = 60%, *p* < 0.0001] and the acupuncture with CHM group [SMD = 4.59, 95% CI (1.53, 7.65), *I* ^2^ = 98%, *p* = 0.003] exhibited greater efficacy in lowering FSH levels compared with the non-acupuncture group (**Figure 11**). However, for the LH levels, no decrease in heterogeneity was observed during the subgroup analysis (**Figure 12**).

#### 3.5.2 Estradiol levels

**Figure 13:**
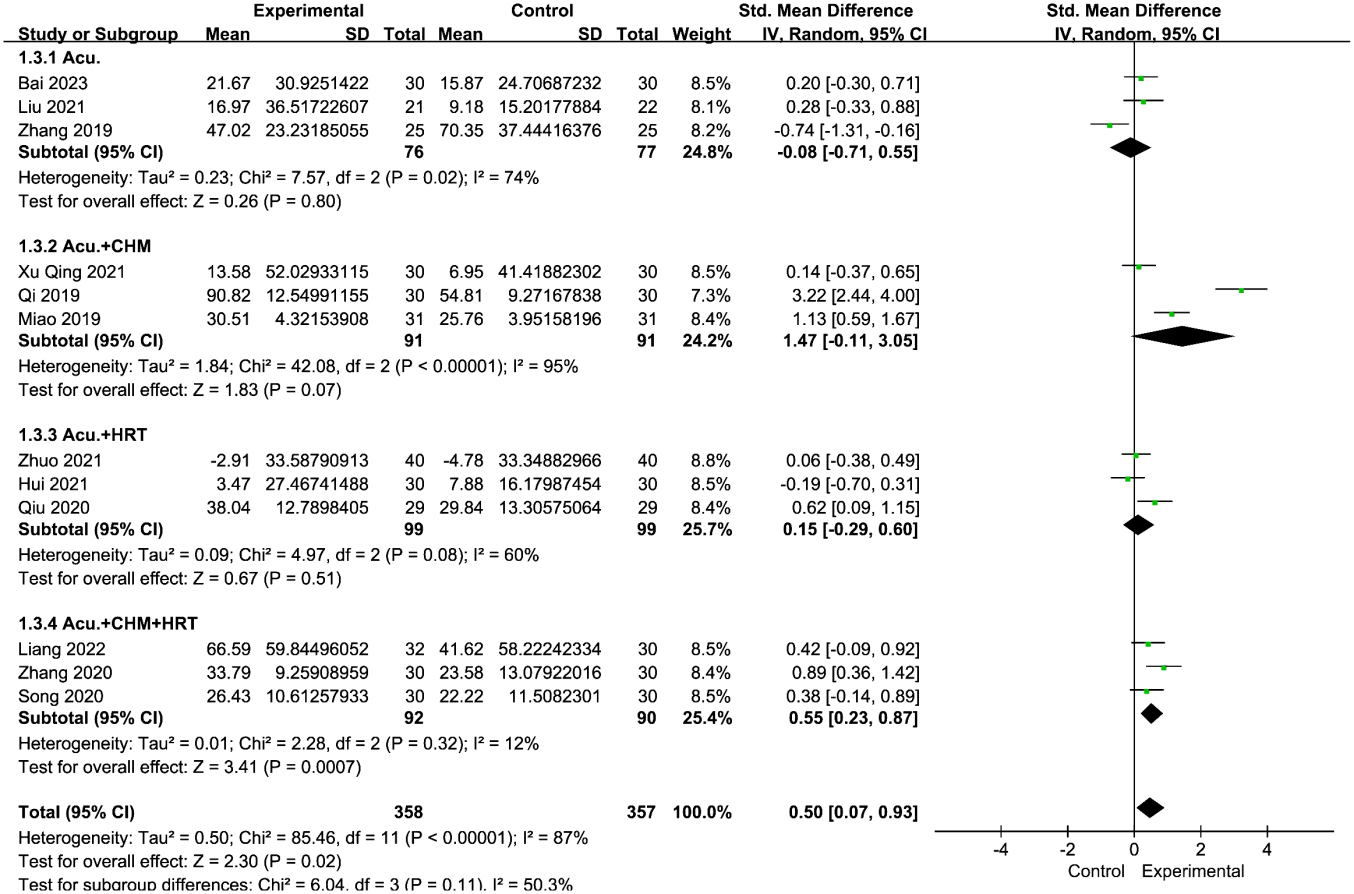
Forest plot illustrating the relationship between E_2_ levels and acupuncture therapy with subgroup analysis.

The meta-analysis indicated that the acupuncture with CHM and HRT group outperformed the control groups [SMD = 0.55, 95% CI (0.23, 0.87), *I* ^2^ = 12%, *p* = 0.0007] (**Figure 13**). High-level heterogeneity was seen in the remaining three subcategories, including nine journals. Rather than using a meta-analysis, a qualitative description was employed. Out of the nine investigations, three (28–30) demonstrated an increase in estradiol levels of the trial groups following treatment, surpassing those of the control groups (*p* < 0.05). The remaining six (26, 27, 35–38) investigations revealed no difference between the two groups (*p* > 0.05).

### 3.6 Publication bias

**Figure 14:**
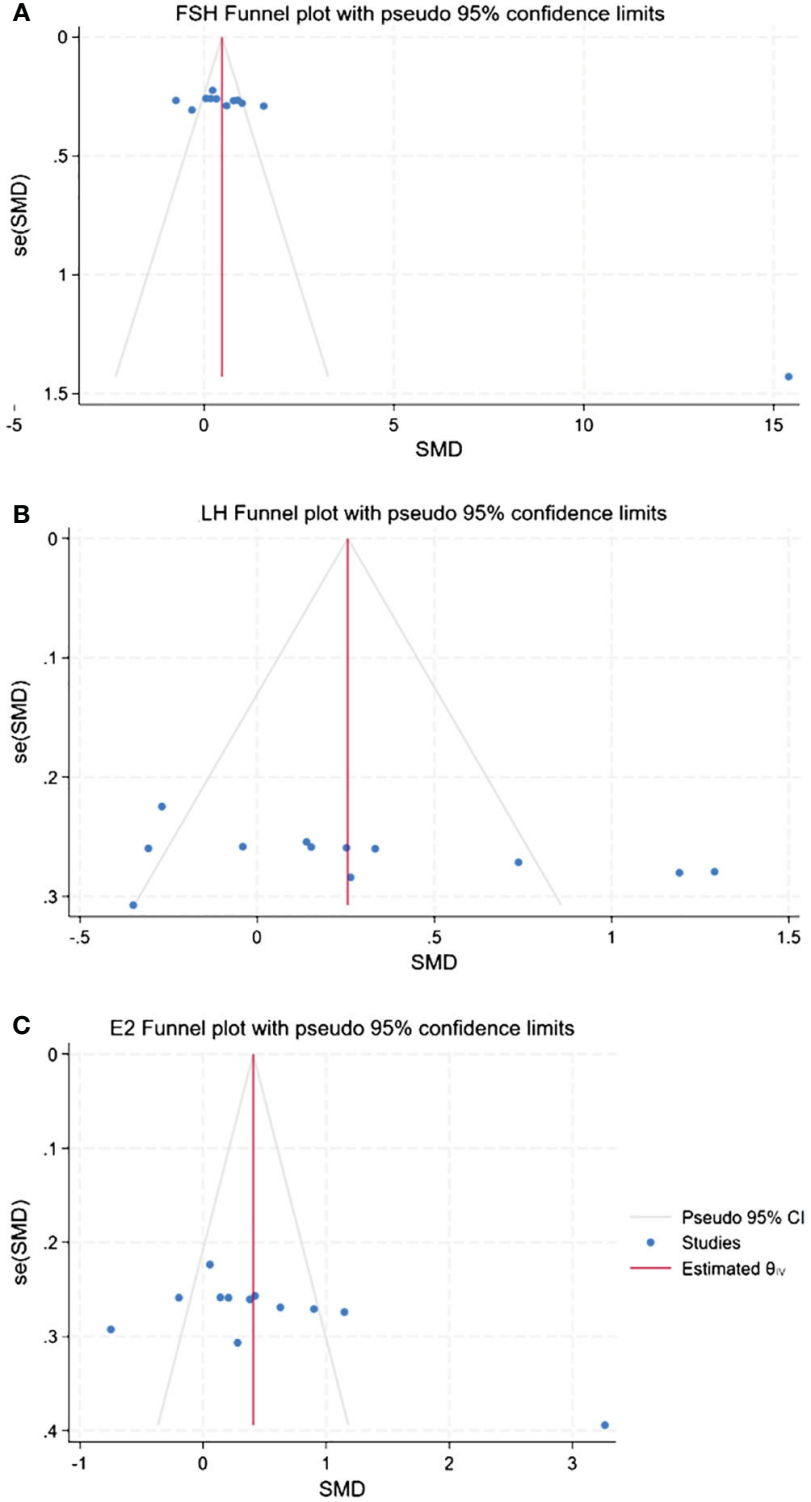
Funnel plots of publication bias. **(A)** Publication bias of FSH. **(B)** Publication bias of LH. **(C)** Publication bias of E_2_.

The presence of publication bias was assessed using Begg’s tests, with a minimum of 10 studies being included in the analysis. There was no apparent asymmetry in the funnel plots, as shown in **Figure 14**. Begg’s test for FSH (*p* = 0.06), LH (*p* = 0.06), and E_2_ (*p* = 0.09) was not significant for publication bias in this meta-analysis due to *p >*0.05.

## 4 Discussion

This study represents the inaugural meta-analysis evaluating the clinical efficacy of acupuncture for POI, employing FSH >25 IU/L as the diagnostic criterion. In this investigation, we incorporated a total of 13 RCTs, involving 775 patients, to scrutinize the efficacy of acupuncture for POI. The findings are succinctly presented as follows: 1) sex hormones—acupuncture demonstrated a significant reduction in FSH levels and an increase in AMH and E_2_ levels in POI patients, with negligible impact on LH levels. 2) Follicular development status—following the reduction of heterogeneity, acupuncture yielded a significant improvement in AMH levels, while no discernible difference was observed in AFC. 3) Climacteric symptoms—patients subjected to acupuncture exhibited a higher overall effective rate compared with their counterparts without acupuncture. However, the acupuncture group manifested no improvement in the Kupperman index, and the difference in adverse effects between the two groups lacked statistical significance.

FSH possesses the capability to enhance follicular development and stimulate estrogen secretion. Elevated FSH levels are associated with excessive follicular depletion, culminating in diminished follicular reserve. Lower FSH levels can enhance fertility probabilities. Serving as a biomarker of follicular development, estrogen not only protects the cardiovascular system and nerves but also prevents the risk of bone rarefaction. Addressing estrogen deficiency effectively markedly improves the quality of life for POI patients. AMH can prevent premature follicular depletion by inhibiting the recruitment of primordial follicles. According to our findings, acupuncture demonstrated the potential to lower FSH levels, elevate E_2_ and AMH levels, and alleviate associated symptoms. Preantral follicle development took 85 days to mature, during which time the follicles underwent continuous growth (grades 1 to 4) and exponential growth (grades 5 to 8). The findings of the study suggest that acupuncture has the potential to decrease FSH levels, increase E_2_ and AMH levels, and alleviate associated symptoms. However, due to the long time required for follicle development from the preantral follicle to the antral follicle, which can be observed by B-ultrasound, this study did not find any benefit of acupuncture treatment on antral follicle-related outcomes.

In contrast to previous systematic reviews, we adhered to the latest diagnostic criteria for POI as outlined by the ESHRE. Additionally, the intervention window was advanced. The literature incorporated into this study was not considered in prior relevant articles. Consistent with earlier discoveries (40, 41), our findings demonstrate the efficacy of acupuncture in lowering serum FSH levels and increasing E_2_ levels. Limited research has focused on the isolated effects of acupuncture. Our study revealed that acupuncture has the potential to elevate AMH levels and the overall effective rate, exerting positive effects on menstrual disorders and perimenopausal symptoms.

Acupuncture, emerging as a novel therapy for POI, has been demonstrated to modulate a diverse array of cellular processes and pathways. In another investigation, acupuncture exhibited the capacity to stimulate Bcl-2 and diminish Bax expression in ovarian tissues, thereby mitigating granulosa cell apoptosis and impeding primordial follicle loss through the induction of antioxidant and anti-apoptotic systems (42). Additionally, Zhang et al. (43) unveiled that electroacupuncture could potentially inhibit the phosphorylation of proteins in the PI3K/AKT/mTOR pathway, leading to the restoration of serum levels of AMH, E_2_, FSH, and LH, along with the proliferation of small follicles. Remarkably, electroacupuncture exhibited the potential to regulate characteristic metabolites linked to energy and neurotransmitter metabolism in the liver and kidney, thereby enhancing the menstrual cycle in the participants (44). Moreover, a recent experiment revealed that electroacupuncture could potentially stabilize hormone levels and mitigate follicular atresia by upregulating the expression of CDK6/CCND1 in murine ovarian granulosa cells (OGCs) (45).

The limitations of this systematic study are as follows: firstly, the clinical effect is susceptible to various factors such as acupoint selection, stimulation intensity, qi generation, and acupuncture techniques, lacking uniformity and objectivity at present. It may contribute to the observed heterogeneity, and no significant improvement was achieved in the subgroup analysis. Furthermore, due to limitations in the source studies, pregnancy outcomes cannot be assessed though serving as a crucial concern for reproductive-aged women with POI. Individuals with POI may experience anxiety, depression, and dyssomnia (46). Although acupuncture has demonstrated benefits for ovarian function, its impact on physical and psychological health or quality of life remains unexplored. Additionally, the thickness of the endometrium should be monitored in time after the rise of estrogen, and if necessary, progesterone should be used to transform the endometrium. Moreover, a majority of the included studies lacked explicit descriptions of double-blind procedures, resulting in methodological shortcomings. Nevertheless, the nature of acupuncture involves mutual interaction between the physician and the patient, making the application of a blinding approach challenging. Therefore, our study represents the inaugural meta-analysis and systematic review of RCTs adhering to the 2016 ESHRE criteria, with a specific focus on acupuncture for POI patients. Despite these limitations, future efforts should involve larger and higher-quality RCTs incorporating essential acupuncture outcomes to comprehensively assess its current clinical efficacy.

## 5 Conclusion

Considering the research findings, acupuncture emerges as a potentially efficacious alternative therapy for the treatment of POI, particularly in light of the existing deficiencies in HRT. However, the quality of evidence remains constrained due to the significant heterogeneity and the small sample effect. Future research is imperative to substantiate the efficacy of acupuncture in the treatment of POI, necessitating meticulously designed, large-scale and multicenter RCTs.

## Data availability statement

The original contributions presented in the study are included in the article/**Supplementary Material**. Further inquiries can be directed to the corresponding author.

## Author contributions

HC: Conceptualization, Data curation, Formal analysis, Investigation, Methodology, Software, Writing – original draft, Writing – review & editing. HL: Conceptualization, Data curation, Formal analysis, Investigation, Methodology, Software, Writing – original draft, Writing – review & editing. GL: Conceptualization, Data curation, Formal analysis, Investigation, Methodology, Software, Writing – review & editing. XL: Conceptualization, Data curation, Formal analysis, Investigation, Methodology, Software, Writing – review & editing. SL: Project administration, Supervision, Validation, Writing – review & editing. PL: Conceptualization, Methodology, Validation, Writing – review & editing. CC: Project administration, Supervision, Validation, Writing – review & editing. LX: Project administration, Resources, Supervision, Validation, Writing – review & editing.
