# Supplementary material for: The clinical value of acupuncture for women with premature ovarian insufficiency: a systematic review and meta-analysis of randomized controlled trials
Source: Front Endocrinol (Lausanne). 2024 Jul 11;15:1361573. doi: 10.3389/fendo.2024.1361573 (PMC11269250; doi:10.3389/fendo.2024.1361573)

Supplement:Data sheet

1. **Supplementary Data**

**1).Pubmed**

**Search Date: October 07, 2023**

((((Premature Menopause[Title/Abstract]) OR ("Menopause, Premature"[Mesh])) OR ((((((((premature ovarian insufficiency[Title/Abstract]) OR (premature ovarian failure[Title/Abstract])) OR (primary ovarian failure[Title/Abstract])) OR (ovarian insufficiency[Title/Abstract])) ) OR (Familial premature ovarian failure[Title/Abstract])) OR ("Primary Ovarian Insufficiency"[Mesh])) OR ("Premature ovarian failure, familial" [Supplementary Concept]))) AND ((((((((((((((((("Acupuncture"[Mesh]) OR ("Acupuncture Therapy"[Mesh])) OR (pharmacopuncture[Title/Abstract])) OR (acupuncture treatment[Title/Abstract])) OR (acupuncture treatments[Title/Abstract])) OR (treatment, acupuncture[Title/Abstract])) OR (therapy, acupuncture[Title/Abstract])) OR (pharmacoacupuncture treatment[Title/Abstract])) OR (treatment, pharmacoacupuncture[Title/Abstract])) OR (pharmacoacupuncture therapy[Title/Abstract])) OR (therapy, pharmacoacupuncture[Title/Abstract])) OR (acupotomy[Title/Abstract])) OR (acupotomies[Title/Abstract])) OR (needles[Title/Abstract])) OR (needling[Title/Abstract])) OR (needle therapy[Title/Abstract])) OR (warming needle[Title/Abstract]))) AND (Randomized controlled trial[Publication Type] OR randomized[Title/Abstract] OR placebo[Title/Abstract])


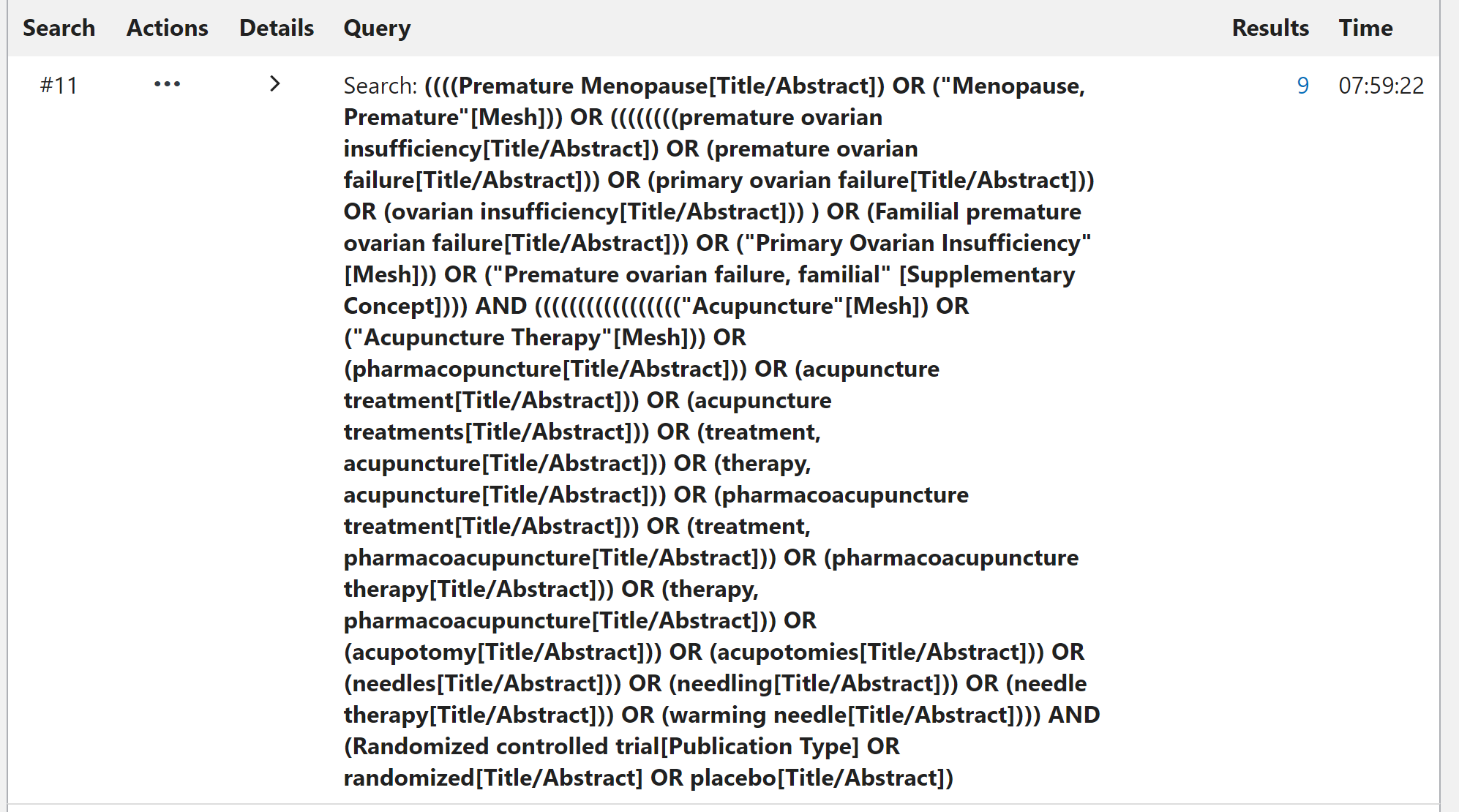


**Result: 31 papers**

**2). Cochrane library**

**Search Date: October 07, 2023**


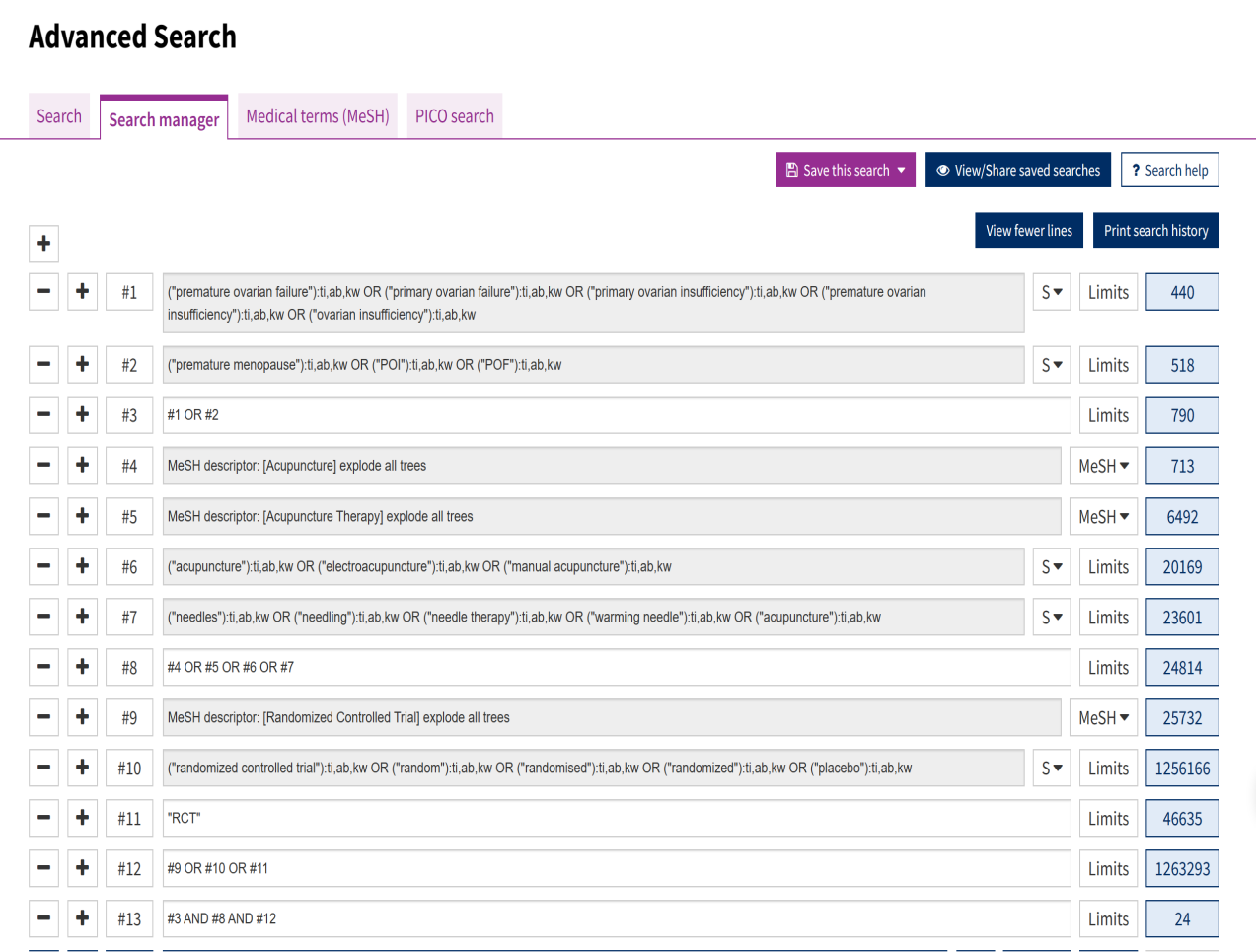


**Result: 24 papers**

**3).Web of sci**

**Search Date: October 07, 2023**

((((((TS=(premature ovarian insufficiency)) OR TS=(premature ovarian failure)) OR TS=(primary ovarian failure)) OR TS=(ovarian insufficiency)) OR TS=(premature menopause)) OR TS=(menopause, premature)) OR TS=(primary ovarian insufficiency) and Preprint Citation Index (Exclude – Database)

(((((((((TS=(acupuncture)) OR TS=(acupuncture therapy)) OR TS=(electroacupuncture)) OR TS=(needles)) OR TS=(needling)) OR TS=(needle therapy)) OR TS=(warming needle)) OR TS=(manual acupuncture)) OR TS=(acupuncture treatment)) OR TS=(acupotomy) and Preprint Citation Index (Exclude – Database)

(((TS=(randomized controlled trial)) OR TS=(randomized)) OR TS=(random)) OR TS=(placebo) and Preprint Citation Index (Exclude – Database)


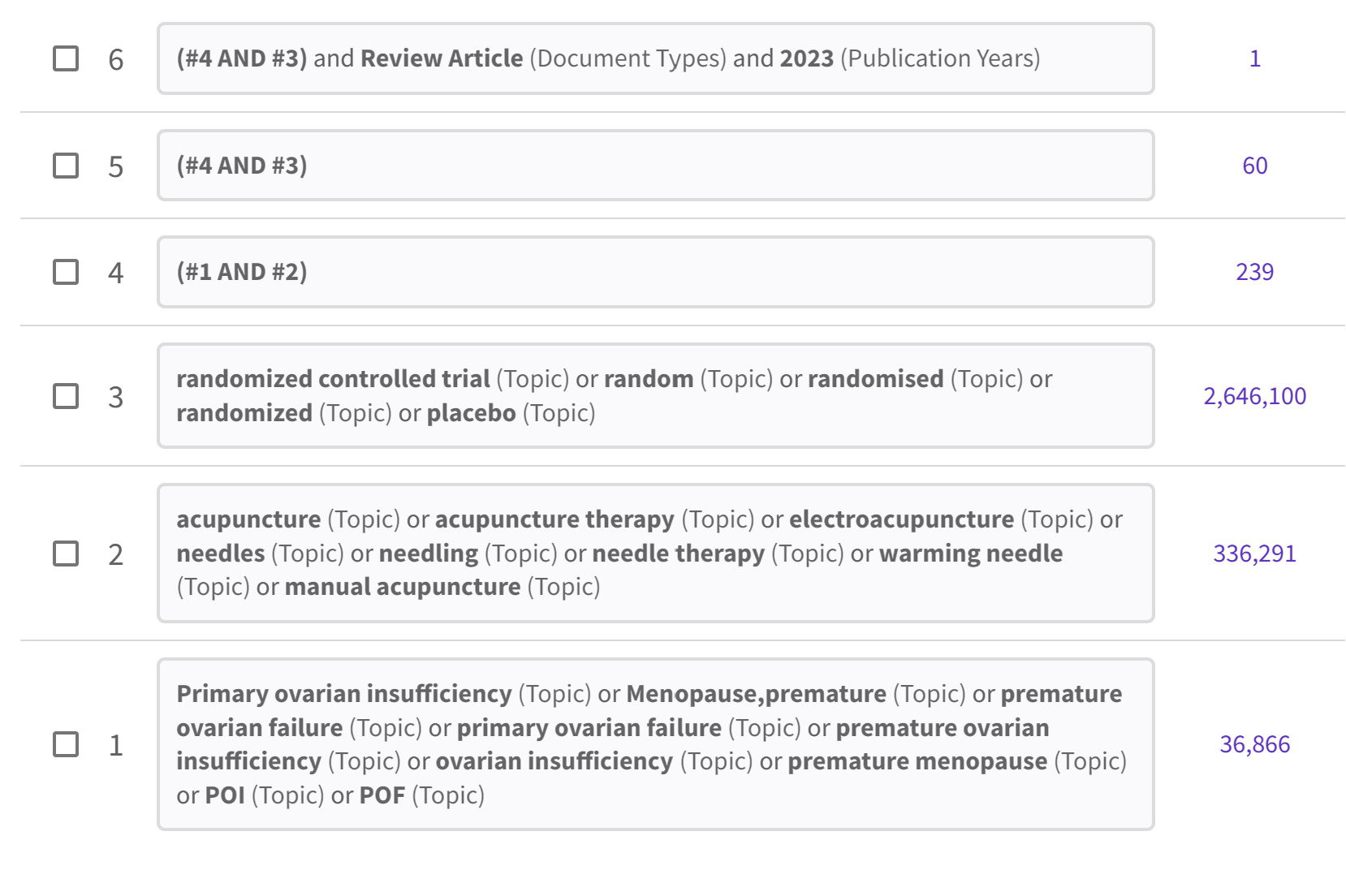


**Result: 60 papers**

**4). CNKI database**

**Search Date: October 07, 2023**


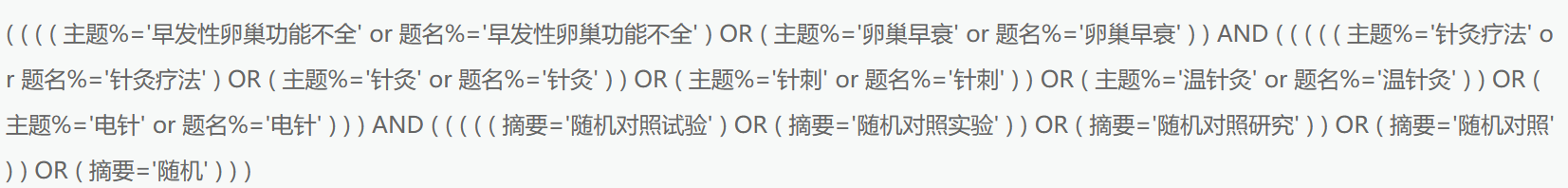


**Result: 157 papers**

**5). Wanfang database**

**Search Date: October 07, 2023**

主题:(早发性卵巢功能不全 OR 卵巢早衰) and 主题:(针灸疗法 OR 针刺 OR 电针 OR 温针灸 OR 针灸) and 主题:(随机对照实验 OR 随机对照试验 OR 随机对照研究 OR 随机 OR RCT OR 随机对照)

**Result: 162 papers**

**6). CBM database**

**Search Date: October 07, 2023**

(("随机对照实验"[常用字段:智能] OR "随机对照研究"[常用字段:智能] OR "随机对照"[常用字段:智能] OR "随机"[常用字段:智能] OR "RCT"[常用字段:智能]) OR ("随机对照试验"[不加权:扩展])) AND (((("针刺"[常用字段:智能] OR "针灸"[常用字段:智能] OR "电针"[常用字段:智能] OR "温针灸"[常用字段:智能])) OR ("针灸疗法"[不加权:扩展]))) AND (((早发性卵巢功能不全) OR (卵巢早衰)))


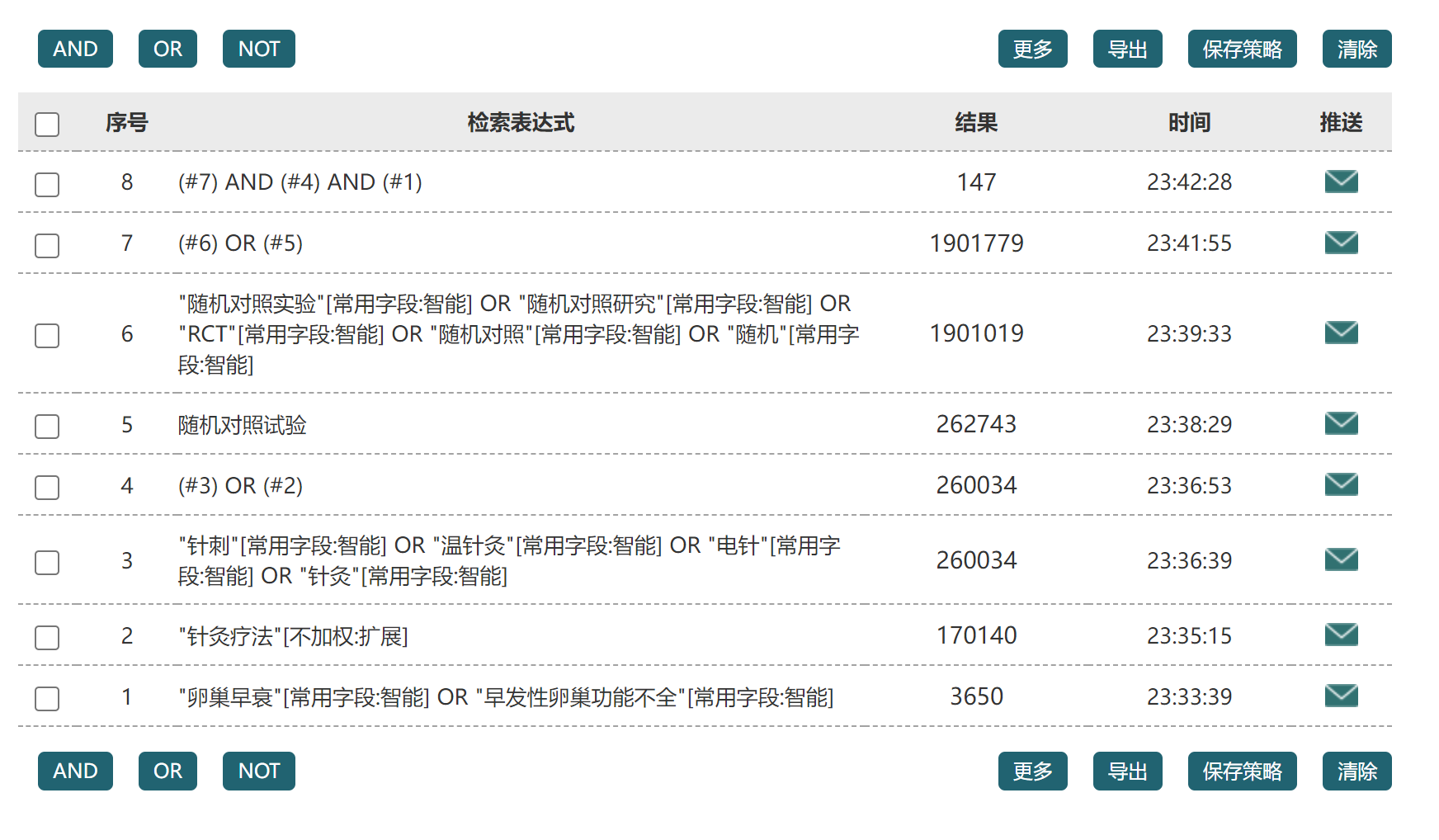


**Result: 147 papers**

**7).CQVIP database**

**Search Date: October 07, 2023**


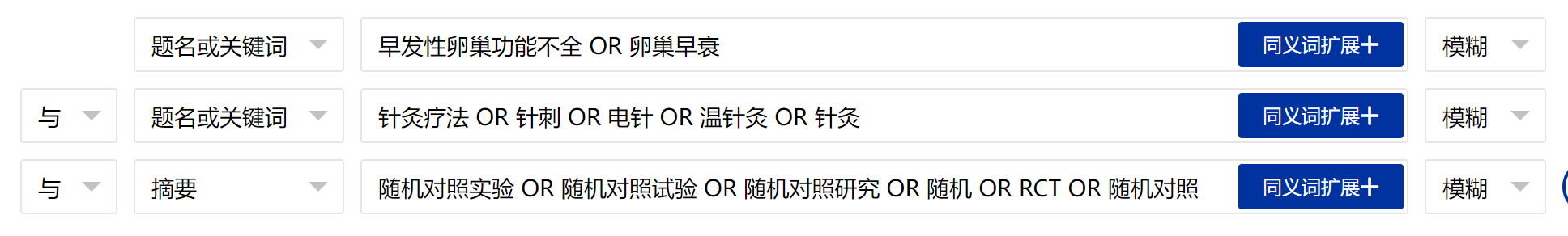


**Result: 106 papers**

**8).Embase database**

**Search Date: October 07, 2023**


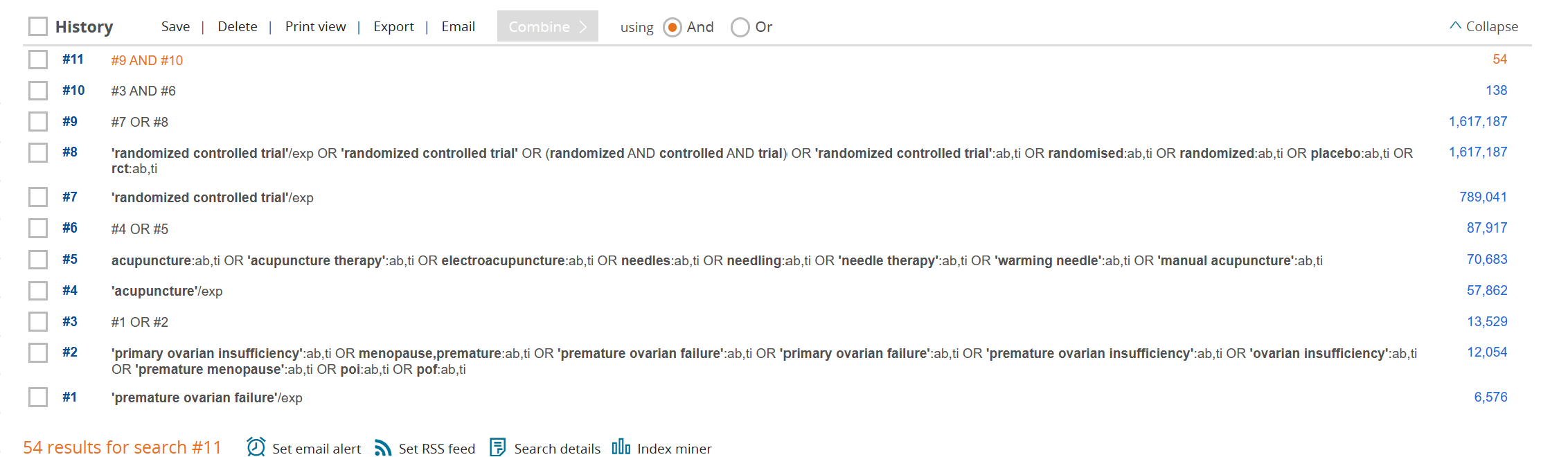


**Result: 54 papers**

**Total: 741 papers**

1. **Supplementary Figures and Tables**

The total effective rate was based on the score scale of the menstrual cycle, menstrual blood volume, and the severity of the common symptoms in patients with POI, such as palpitations, insomnia, hot flashes, and sweating etc. Scores were performed before and after treatment, and the improvement of symptoms was greater than or equal to 30%, which was considered effective.The table below shows the rating scale used in one of the studies.


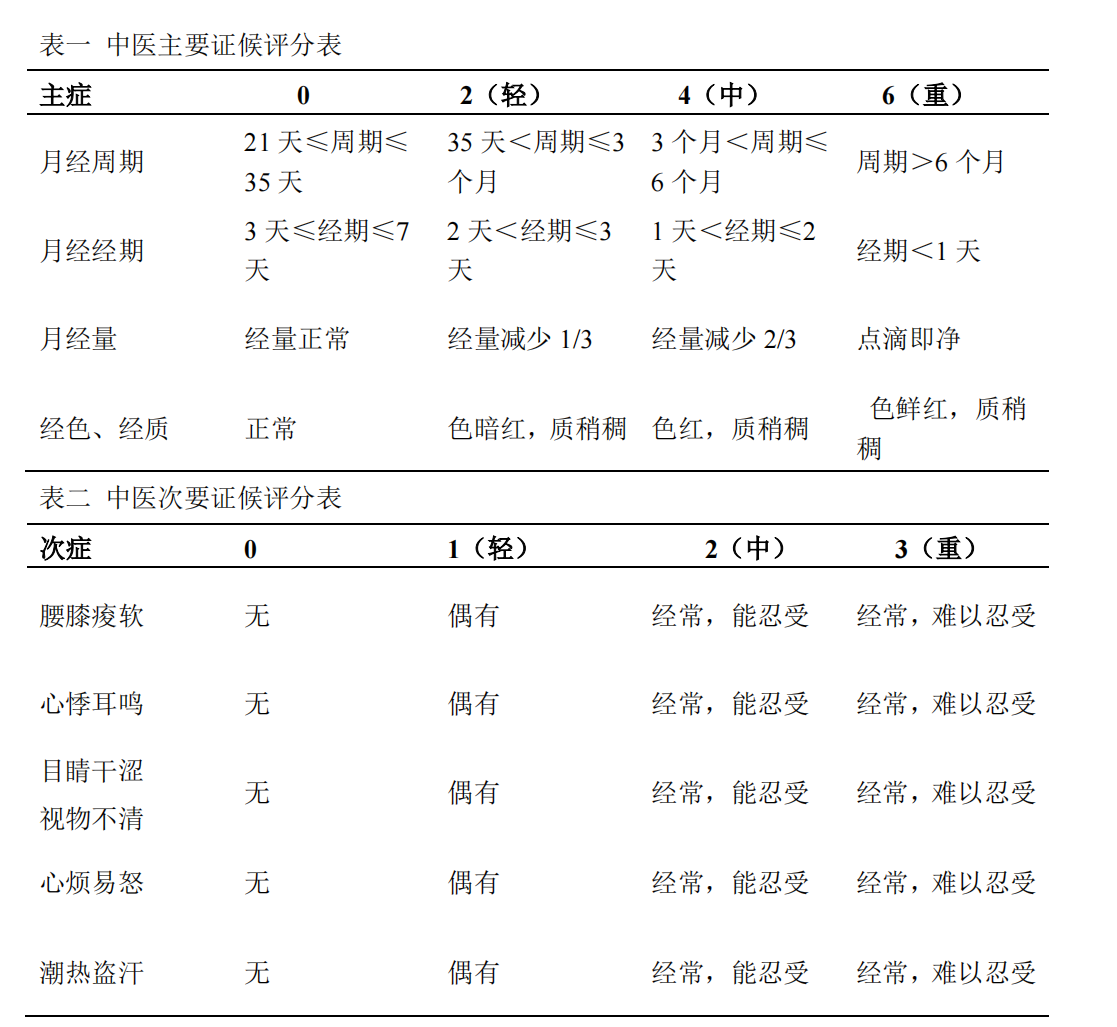

Supplement: Supplementary file 1 [file DataSheet_1.docx]
